# Psychometric validation of Utrecht Work Engagement Scale-Student-9 in Chinese undergraduate students

**DOI:** 10.3389/fpsyg.2025.1486363

**Published:** 2025-09-15

**Authors:** Zhixia Wei, Tiantian Yang, Xiaoxia Gu, Jingyi Dong, Norlizah Che Hassan

**Affiliations:** ^1^Hebei Key Laboratory of Children's Cognition and Digital Education, School of Educational Studies, Langfang Normal University, Langfang, Hebei, China; ^2^Section of Foreign Cooperation and Exchange Office for International Exchange and Cooperation, Yunnan Minzu University, Kunming, Yunnan, China; ^3^School of Public Administration, Nanfang College, Guangzhou, Guangdong, China; ^4^Faculty of Educational Studies, Universiti Putra Malaysia, Serdang Selangor, Selangor, Malaysia

**Keywords:** academic engagement, undergraduate students, psychometric validation, UWES-S-9, bifactor-ESEM model

## Abstract

**Introduction:**

Academic engagement represents a critical construct in educational psychology, yet comprehensive psychometric validation of assessment instruments remains limited within Chinese higher education contexts. This study examines the psychometric properties of the Chinese version of the Utrecht Work Engagement Scale-Student-9 (UWES-S-9) among undergraduate students.

**Methods:**

Participants comprised 498 Chinese undergraduate students (66.1% female; Mage = 19.15 years, SD = 1.03, range: 17–24) who completed the UWES-S-9. A comprehensive analytical approach was employed, including confirmatory factor analysis (CFA), exploratory structural equation modeling (ESEM), and bifactor modeling (CFA and ESEM), alongside assessments of internal consistency, test-retest reliability, construct and concurrent validity, and measurement invariance across gender, academic year, and major.

**Results:**

The three-factor ESEM model demonstrated superior fit indices (χ^2^/df = 2.899, RMSEA = 0.062, CFI = 0.990, TLI = 0.971) compared to traditional CFA approaches. Bifactor analysis indicated that academic engagement is primarily characterized by a general factor accounting for 79% of common variance. The omega hierarchical coefficient for the general factor (ωH = 0.827) supported reliable interpretation of total engagement scores, while specific factor reliabilities were negligible (ωHS ≤ 0.149), suggesting limited utility of subscale scores. Measurement invariance testing supported configural, metric, and scalar invariance across gender, major, and academic year.

**Discussion:**

These findings contribute to the validation of the UWES-S-9 within Chinese higher education contexts while providing evidence for predominantly unidimensional interpretation of academic engagement scores. The results suggest that total engagement scores provide more reliable assessment than individual subscale scores, with important implications for both research and practical applications in Chinese educational settings.

## 1 Introduction

Academic engagement is an important predictor of students' academic performance and mental health status, and it has attracted widespread attention along with the development of positive psychology (Meng and Jin, 2017). Only when students are actively engaged in academics can they learn better. The concept of academic engagement evolved from “engagement,” which is the extension of work engagement in the field of education. Initial research conceptualized academic engagement as a unidimensional construct (Epstein and McPartland, 1976). As studies progressed, a multidimensional understanding of academic engagement gained traction among researchers. (Schaufeli et al. 2002b) proposed a three-dimensional model of academic engagement consisting of vigor, dedication, and absorption. They defined academic engagement as the positive, enthusiastic learning attitude, abundant energy, and deep immersion displayed by individuals during the learning process. Nevertheless, the structural dimensions of academic engagement continue to be the focus of considerable scholarly debate. Perspectives that view academic engagement as a holistic phenomenon persist (Dominguez-Lara et al., 2025; Salamon et al., 2021), and this divergence is reflected not only at the conceptual level, but also in its measurement.

In terms of measurement of academic engagement, (Schaufeli et al. 2002b) compiled the Utrecht Work Engagement Scale-Student (UWES-S) based on the Work Engagement Scale (UWES) and took college students as samples (Schaufeli et al., 2002a,b). The three-factor structure of UWES-S with 17 items has been validated across various cultures through numerous empirical studies (Meng and Jin, 2017; Wickramasinghe et al., 2018), demonstrating its cross-cultural stability and has been widely adopted for measuring student academic engagement. However, the proliferation of multi-variable surveys often leads to participant fatigue due to numerous items. To address this, a more concise measure that maintains accuracy while reducing response time is desirable, potentially improving participant cooperation. In response to this need, (Schaufeli et al. 2006) developed the Utrecht Work Engagement Scale-9 (UWES-9), a condensed version of the Utrecht Work Engagement Scale (UWES), using a comprehensive international database. This shortened scale, adapted for educational settings as the Utrecht Work Engagement Scale-Student-9 (UWES-S-9), offers a more user-friendly alternative to the longer UWES-S, minimizing unnecessary participant burden.

The UWES-S-9 has undergone validation across various countries, demonstrating robust reliability and validity. However, its structural composition remains a subject of debate. Some studies support a one-factor structure (Chi et al., 2023; Dominguez-Lara et al., 2025, 2020; Römer, 2016; Serrano et al., 2019), while others validate a three-factor model comprising Vigor, Dedication, and Absorption (Carmona-Halty et al., 2019; Loscalzo and Giannini, 2019; Mills et al., 2012; Rastogi et al., 2018; Sulla et al., 2023). Additionally, few researchers derived a two-factor model of UWES-S-9. For instance, (Dimitriadou et al. 2020) identified a two-factor model among Greek university students, wherein Dedication and Absorption were combined into a single factor. Conversely, (Portalanza Chavarría et al. 2017) proposed an alternative two-factor configuration among Ecuadorian higher education students, merging Vigor and Absorption into one factor while retaining Dedication as a separate construct.

The reason for this discrepancy remains elusive and may be attributed to cultural variations in the conceptualization and self-reporting of academic engagement (Dimitriadou et al., 2020). Participants from diverse cultural backgrounds may interpret and express their academic engagement differently, potentially due to varying educational norms, values, and expectations. This observed incongruence underscores the imperative for further cross-cultural research to elucidate the factorial structure of academic engagement across different cultural milieus. Such investigations are crucial for enhancing the validity and reliability of academic engagement measures across diverse populations, thereby contributing to a more nuanced and culturally sensitive understanding of academic engagement in global educational contexts.

Moreover, the discrepancies in the factor structure of the UWES-S-9 may be partly due to methodological issues. Past research on the UWES-S-9 has several methodological shortcomings that may impact the factor structure findings and their interpretation. For instance, the use of confirmatory factor analysis (CFA) alone may not be sufficient to capture the complexity of the construct. CFA assumes that items are perfect indicators of a single factor, which may not be the case in real-world data (Asparouhov and Muthén, 2009; Morin et al., 2016). This can lead to an overestimation of factor correlations and potential biases in the interpretation of the factor structure. Additionally, the reliance on model fit indices alone may not provide a complete picture of the construct's validity. The substantial correlations between the three factors suggest potential redundancy, which may not be adequately addressed by traditional fit indices (Dominguez-Lara et al., 2025). This underscores the need for a more nuanced approach to evaluating the factor structure of the UWES-S-9.

Salamon et al. (2021) used a comprehensive factor-analytic framework, including one-factor, first-order, higher-order, and bifactor confirmatory factor analysis, and found that a bifactor model of UWES-9, which includes a global engagement factor alongside three specific factors, provided the most accurate representation of the data. This suggests that engagement might be experienced as a global phenomenon with specific domains. Additionally, studies using exploratory structural equation modeling (ESEM) and bifactor-ESEM found that the general factor accounted for the overwhelming variance in UWES-S-9 items, implying that the scale is essentially unidimensional (Dominguez-Lara et al., 2020, 2021). Recently, (Dominguez-Lara et al. 2025) conducted a comprehensive evaluation of the psychometric properties of the UWES-S-17 and UWES-S-9 using an integrative factor-analytic approach that incorporated CFA, exploratory structural equation modeling (ESEM), as well as their corresponding bifactor extensions (bifactor-CFA and bifactor-ESEM). Utilizing data from a sample of Ecuadorian university students, the study provided empirical support for the conceptualization of the UWES-S as a predominantly unidimensional construct, challenging previously established multidimensional interpretations prevalent in the literature.

This comprehensive analytical framework offers several advantages over traditional single-method approaches. ESEM's flexibility in allowing cross-loadings provides more realistic modeling of complex psychological constructs and yields less biased factor correlation estimates compared to CFA's restrictive zero cross-loading constraints (Asparouhov et al., 2015). Simultaneously employing both CFA and ESEM enables robust comparisons of parameter estimates and factor correlations, facilitating evidence-based decisions regarding factor distinctiveness (Marsh et al., 2014). The incorporation of bifactor models further enhances this framework by explicitly partitioning variance between general and specific factors, thereby addressing critical questions regarding dimensionality that cannot be resolved through hierarchical models alone (Dominguez-Lara et al., 2025). This approach enables researchers to empirically determine whether the UWES-S-9 is best conceptualized as primarily unidimensional, strictly multidimensional, or as having both strong general and specific components—a distinction with significant implications for scoring practices and theoretical interpretations. Moreover, this integrative approach also enhances measurement precision by accounting for psychometric issues such as item wording effects and offers greater cross-cultural generalizability in instrument validation (DiStefano and Motl, 2006; Lindwall et al., 2012). However, limited studies have employed a comprehensive analytical framework including CFA, ESEM, and bifactor solutions to assess the dimensionality of UWES-S-9 (Dominguez-Lara et al., 2025, 2021). Given that previous validation studies involving Chinese student samples have yielded conflicting conclusions, where some studies found a one-factor structure for the UWES-S-9 scale (Chi et al., 2023) while others identified a three-factor structure (Meng and Jin, 2017). This study seeks to address these inconsistencies by employing a comprehensive and previously unutilized methodological approach in the Chinese context, integrating one-factor CFA, three-factor CFA, three-factor ESEM, and corresponding bifactor models to examine the dimensionality of the UWES-S-9 systematically.

Notably, there is a paucity of systematic validation studies focusing on Chinese undergraduate students from normal universities who receive professional training and want to pursue careers in teaching or related disciplines (Wei et al., 2025). After completing their professional studies, students from normal universities usually need to participate in teacher qualification exams and other related exams and obtain corresponding teacher qualification certificates before engaging in teaching work. As teachers of primary and secondary schools and kindergartens in the future, they shoulder the important task of teaching and disseminating knowledge and play an important role in the education field. Comparable teacher-training institutions exist globally, and this study aims to contribute to the ongoing factor structure debate by providing empirical evidence on the UWES-S-9's factor structure solution within this previously unexplored population.

Importantly, the cross-cultural validation study conducted by (Schaufeli et al. 2002b) did not include Asian countries, and to the best of our knowledge, the scale equivalence assessment of UWES-S-9 using its correlations with the UWES-S has not been tested in current validation studies among other Asian countries (Schaufeli et al., 2006), so the present study aimed to bridge a gap in this area. Additionally, given that previous validation studies of UWES-9 and UWES-S-9 have consistently found negative correlations between engagement and burnout (Rastogi et al., 2018; Römer, 2016; Schaufeli et al., 2006), this study also seeks to examine the relationship between academic engagement and academic burnout to further verify the concurrent validity of the UWES-S-9. Moreover, its other psychometric properties such as the test-retest reliability, and the measurement invariance across gender, academic year, and major are also assessed in this study.

Furthermore, previous studies employing the UWES-S have reported inconsistent findings regarding the influence of demographic variables on academic engagement (Casuso-Holgado et al., 2013; Liébana-Presa et al., 2014; Loscalzo and Giannini, 2019; Mostert et al., 2007). These discrepancies raise concerns about whether observed group differences reflect true variations in academic engagement or are confounded by measurement issues. Without such verification, any observed group differences may stem from differential item functioning rather than substantive psychological differences (Cheung and Rensvold, 2002; Millsap, 2012). However, research examining the measurement equivalence of the UWES-S-9 across demographic variables remains limited in the literature. Notable exceptions include Serrano et al.'s (2019) investigation of measurement invariance across gender and age groups among Spanish secondary school students, and the work of (Carmona-Halty et al. 2019), which established gender-based measurement equivalence within a Chilean undergraduate sample. To address this issue, it is essential to examine the measurement invariance of the UWES-S-9 across key demographic groups, which ensures that the construct is interpreted equivalently across groups, thereby allowing for meaningful and unbiased comparisons (Putnick and Bornstein, 2016; Vandenberg and Lance, 2000). Moreover, invariance testing is a prerequisite for evaluating the effectiveness of interventions targeting academic engagement, as it allows researchers to attribute changes in engagement scores to the intervention itself rather than to inconsistencies in measurement.

Thus, to address the identified research gaps, this study pursued two primary research objectives: (1) Confirm the factor structure of the UWES-S-9 using a comprehensive approach that includes CFA, ESEM, bifactor-CFA, and bifactor-ESEM; (2) Assess other psychometric properties of the UWES-S-9, including internal consistency reliability, test-retest reliability, construct validity, factor distinctiveness, its scale equivalence and concurrent validity by examining its correlations with the original scale and academic burnout, respectively, and its measurement invariance across demographic variables.

## 2 Materials and methods

### 2.1 Participants

The target population of this study is the Chinese undergraduate students studying in normal universities of H province. The sample size should be determined based on the literature and statistical methods used in the study. Considering that the data analysis method used in this study includes CFA, the current study referred to the sample size determination proposed by (Wolf et al. 2013). According to these criteria, the minimum sample size required for a CFA model with three factors and three indicators for each factor should be about 420 to get the factor loadings of 0.5 and this sample size can meet all the priori criteria in the literature. Under this guideline, 515 participants were recruited from three normal universities in H province, who can meet the criteria including no serious physical illness, no diagnosed psychological problems or psychiatric illness.

A total of 498 valid questionnaires were obtained and the age of the participants ranged from 17 to 24 (M = 19.15 ± 1.03; 66.1% females). Descriptive analysis was conducted based on the demographic information of respondents, which reflects the distribution of respondents on different demographic variables. The results show that there were 140 (28.1%) undergraduate students came from the first academic year, and 127 (25.5%), 116 (23.3%), and 115 (23.1%) respondents from the second to the fourth academic year, respectively. Among the participants, 218 (43.8%) were majoring in humanities, 156 (31.3%) participants in science and Engineering, and 124 (24.9%) in arts.

### 2.2 Measures

This study employed the Chinese version of the Utrecht Work Engagement Scale-Student (UWES-S) and its short version (UWES-S-9) to assess academic engagement among undergraduate students. Developed by (Schaufeli et al. 2002a), the UWES-S comprises three subscales: Vigor, Dedication, and Absorption. The 17-item scale utilizes a seven-point Likert format, with responses ranging from 0 (“never”) to 6 (“always/every day”). Higher scores indicate greater academic engagement. In this study, the Chinese version adapted by (Li and Huang 2010) was referred to. The subscales demonstrated good composite reliability with values of 0.838 (Vigor), 0.820 (Dedication), and 0.812 (Absorption) based on the current sample.

The UWES-S-9, a 9-item adaptation of the UWES-9, was tailored for educational contexts (Schaufeli et al., 2006). It maintains the three-subscale structure, with each subscale containing three items. Vigor assesses energy and enthusiasm in learning, Dedication reflects positive emotional experiences from learning, and Absorption measures concentration levels during the study. The Chinese version of the UWES-S-9 used in this study was likewise translated by (Li and Huang 2010).

Academic burnout was evaluated using the Learning Burnout Scale of Undergraduates (LBS), adapted by (Lian et al. 2005) based on the Maslach Burnout Inventory-Student Survey (MBI-SS) (Schaufeli et al., 2002b). This 20-item scale encompasses three dimensions: Low Mood, Inappropriate Behavior, and Low Sense of Accomplishment. Items are rated on a five-point Likert scale from 1 (“Not at all like me”) to 5 (“Very much like me”), yielding total scores between 20 and 100. Higher scores indicate greater academic burnout. The study found good reliability for the LBS subscales, with composite reliability values of 0.831 (Low Mood), 0.785 (Inappropriate Behavior), and 0.756 (Low Sense of Accomplishment).

### 2.3 Procedure

This research received ethical approval from the Ethics Committee for Research involving Human Subjects of University Putra Malaysia Research (NO: JKEUPM-2023-137). After that, a pilot study (n=203) was conducted to check if there was any misunderstanding among the participants toward the items. It was found that all the items can be understood clearly and there was no ambiguous expression. Two weeks later, to evaluate the test-retest reliability of the questionnaire, the respondents who participated in the pilot study were retested (n=203). Finally, the electronic version of the questionnaire was distributed to the participants through the Questionnaire Star platform and 515 respondents participated in the actual study.

### 2.4 Data analysis

After data collection, SPSS 26.0 was used for data examination, descriptive statistical analysis, and reliability tests. Subsequently, multiple competing measurement models were systematically examined using Mplus 8.3 to determine the optimal factorial representation of the UWES-S-9.

The analysis proceeded through four distinct phases: (1) confirmatory factor analysis (CFA) comparing one-factor and three-factor models, (2) exploratory structural equation modeling (ESEM) to address potential model misspecification, (3) bifactor-CFA incorporating both general and specific factors, and (4) bifactor-ESEM combining the advantages of both bifactor modeling and ESEM approaches.

The one-factor model specified all nine items loading onto a single latent construct representing overall academic engagement. The three-factor model posited items loading onto three correlated dimensions—Vigor, Dedication, and Absorption—consistent with previous theoretical expectations. The ESEM models allowed for cross-loadings between factors while maintaining the target factor structure. Bifactor models incorporated both a general engagement factor and three specific factors (Vigor, Dedication, Absorption) to examine the extent to which items reflected general vs. specific variance components.

Model adequacy was assessed using multiple complementary approaches. Conventional goodness-of-fit indices included the chi-square to degrees of freedom ratio (χ^2^/df), root mean square error of approximation (RMSEA) with 90% confidence intervals, comparative fit index (CFI), Tucker-Lewis index (TLI), and standardized root mean square residual (SRMR). Acceptable model fit was indicated by CFI and TLI values ≥ 0.90, RMSEA ≤ 0.08, χ^2^/df ≤ 5.0, and SRMR ≤ 0.08 (Hu and Bentler, 1999; Tabachnick et al., 2013). Model comparison was further informed by information criteria, specifically the Akaike Information Criterion (AIC) and Bayesian Information Criterion (BIC), with lower values indicating superior balance between goodness of fit and parsimony (Vrieze, 2012).

For bifactor models, additional complementary indices were examined to evaluate the appropriateness of the bifactor solution. These included explained common variance (ECV), percentage of uncontaminated correlations (PUC), omega hierarchical coefficients for the general factor (ωH), and omega hierarchical coefficients for specific factors (ωHS). ECV values above 0.70 suggest that the general factor accounts for most common variance, while PUC values above 0.80 are generally preferred, though moderate values (0.50–0.70) can be acceptable in models with multiple specific factors (Rios and Wells, 2014). Omega hierarchical coefficients (ωH) above 0.70 for the general factor support reliable interpretation of total scores, while low ωHS values (< 0.30) indicate limited reliable variance in specific factors beyond the general factor (Smits et al., 2015).

Internal consistency was evaluated using multiple indices appropriate for different factorial structures. McDonald's omega coefficients were computed as more appropriate reliability estimates for multifactor structures, with the threshold of ≥ 0.70 (Hayes and Coutts, 2020). For bifactor models, omega hierarchical coefficients were calculated separately for general and specific factors to assess the reliability of total scores vs. subscale scores. Composite reliability (CR) was also examined, with values ≥ 0.60 considered adequate (Fornell and Larcker, 1981). Corrected item-total correlations above 0.30 were considered indicative of adequate item contribution (Artanti et al., 2018). Test-retest reliability was evaluated using intraclass correlation coefficients (ICCs), with values ≥ 0.75 reflecting good stability and values between 0.50 and 0.75 indicating moderate stability (Koo and Li, 2016).

Factor distinctiveness between UWES-S-9 dimensions was evaluated through examination of factor correlations, average variance extracted (AVE), and shared variance between factors (Fornell and Larcker, 1981). Standard factor loadings were required to exceed 0.50 and remain below 1.0 (Hair et al., 2010). Factor distinctiveness validity was supported when the AVE of each factor exceeded the shared variance with other factors, indicating that factors capture more unique variance than shared variance with other constructs.

Maximum Likelihood Estimation was used in the analysis since the collected data met the normality assumptions of parametric tests (van Zyl and ten Klooster, 2022). The correlation relationship between UWES-S-9 and its original scale (UWES-S) as well as its relationship with academic burnout was tested through bivariate correlations. A sequential multigroup CFA approach was implemented following established methodological procedures to assess the measurement invariance of the UWES-S-9 across gender, academic year, and major (Chen, 2007; Kang et al., 2016). The measurement invariance assessment proceeded through three hierarchical levels of constraint. Initially, configural invariance was examined by simultaneously estimating the baseline model across gender, academic year, and major without imposing equality constraints, thereby testing whether the factor structure remained consistent across groups. Subsequently, metric invariance was assessed by constraining factor loadings to be equal across groups while allowing intercepts and residual variances to vary freely, examining whether the relationship between latent factors and observed indicators remained equivalent. Finally, scalar invariance was evaluated by imposing additional equality constraints on item intercepts across groups. Model comparison was conducted using chi-square difference tests alongside practical fit indices, with invariance supported when changes in model fit indices (ΔRMSEA, ΔSRMR, ΔCFI, ΔTLI, and ΔMNCI) did not exceed the established cutoff criteria of 0.015, 0.03, 0.01, 0.008, and 0.02, respectively (Chen, 2007; Kang et al., 2016).

## 3 Results

### 3.1 Preliminary analyses

Comprehensive data screening procedures were implemented to ensure data quality and statistical assumptions for confirmatory factor analysis. The online survey platform prevented incomplete submissions, resulting in no missing values. However, 15 cases were excluded based on cutoff values (Collier, 2020; Lanning, 1989; Tabachnick et al., 2013): 3 participants with insufficient response time (< 2 min), 2 cases with invariant response patterns (SD < 0.50); 4 univariate outliers (*z*-scores exceeding ±3.29), and 8 multivariate outliers (Mahalanobis d^2^ with *p*1 and *p*2 < 0.001). The final sample comprised 498 participants (96.70% valid response rate). Normality assessment revealed skewness (0.138–1.052) and kurtosis (0.865–1.738) coefficients within acceptable ranges (Collier, 2020; Tabachnick et al., 2013), confirming distributional assumptions for subsequent analyses.

### 3.2 Factor structure of the UWES-S-9

#### 3.2.1 CFA and ESEM models

##### 3.2.1.1 Model comparison and fit assessment

The CFA and ESEM approaches were employed to examine the factorial structure of the UWES-S-9 scale using the data from the actual study, which was comprised of 498 participants. As presented in Table 1, the one-factor CFA model (Model 1) demonstrated poor fit to the data, with χ^2^/df = 10.826, RMSEA = 0.140 [90% CI (0.126–0.155)], CFI = 0.886, TLI = 0.848, and SRMR = 0.051. The information criteria were AIC = 10191.505 and BIC = 10305.191. These indices substantially exceeded acceptable thresholds, indicating inadequate model specification.

**Table 1:** Comparison of model fit indices for the CFA and ESEM models (*n* = 498).

**Models**	**χ^2^/df**	**RMSEA (90% CI)**	**CFI**	**TLI**	**SRMR**	**AIC**	**BIC**
Model 1	10.826	0.140 [0.126–0.155]	0.886	0.848	0.051	10191.505	10305.191
Model 2	4.490	0.084 [0.068–0.100]	0.964	0.946	0.037	10012.964	10139.282
Model 3	2.899	0.062 [0.038–0.086]	0.990	0.971	0.014	9963.986	10140.831

Model 1, One-factor CFA Model; Model 2, Three-factor CFA Model; Model 3, Three-factor ESEM Model.

In contrast, the three-factor CFA model (Model 2) exhibited markedly improved fit statistics: χ^2^/df = 4.490, RMSEA = 0.084 [90% CI (0.068–0.100)], CFI = 0.964, TLI = 0.946, and SRMR = 0.037. The information criteria showed improvement with AIC = 10012.964 and BIC = 10139.282. While representing a considerable enhancement over the unidimensional structure, several indices remained marginally acceptable, suggesting potential model misspecification.

The three-factor ESEM model (Model 3) demonstrated superior fit across all examined indices: χ^2^/df = 2.899, RMSEA = 0.062 [90% CI (0.038–0.086)], CFI = 0.990, TLI = 0.971, and SRMR = 0.014. The information criteria further supported this model's superiority with the lowest values: AIC = 9963.986 and BIC = 10140.831. These values collectively indicated excellent model fit, with the RMSEA falling within the acceptable range and both CFI and TLI exceeding 0.95, suggesting that the ESEM approach better accommodated the complexity inherent in the UWES-S-9 factorial structure.

##### 3.2.1.2 Latent factor correlations and factor distinctiveness

Table 2 presents the latent factor correlations and discriminant validity assessment for both CFA and ESEM models. The three-factor CFA model revealed substantial interfactor correlations, with coefficients of 0.868 between Vigor and Dedication, 0.776 between Vigor and Absorption, and 0.763 between Dedication and Absorption. These correlations substantially exceeded the recommended threshold of 0.70, indicating potential discriminant validity concerns (Byrne et al., 2016).

**Table 2:** Latent correlations and factor distinctiveness for the CFA and ESEM models (*n* = 498).

**Models**	**Latent factor correlations**	**AVE**	**φ**
Model 2	0.868, 0.776, 0.763	VI = 0.574, DE = 0.553, AB = 0.662	VI -DE = 0.754, VI -AB = 0.602, DE-AB = 0.582
Model 3	0.725, 0.605, 0.653	VI = 0.370, DE = 0.462, AB = 0.522	VI -DE = 0.526, VI -AB = 0.366, DE-AB = 0.426

Model 2, Three-factor CFA Model; Model 3, Three-factor ESEM Model; φ, shared variance between two factors; VI, Vigor factor; DE, Dedication factor; AB, Absorption factor.

The AVE values for the CFA model were: Vigor = 0.574, Dedication = 0.553, and Absorption = 0.662. The shared variance (φ) between factors ranged from 0.582 to 0.754. Specifically, the shared variance between Vigor and Dedication (0.754) exceeded both factors' AVE values, and the shared variance between Dedication and Absorption (0.582) exceeded Dedication's AVE (0.553). However, the shared variance between Vigor and Absorption (0.602) did not exceed Absorption's AVE (0.662) but was comparable to Vigor's AVE (0.574). This pattern suggests inadequate factor distinctiveness, particularly between Vigor-Dedication and Dedication-Absorption factor pairs. The results have implications for whether these dimensions should be treated as separate scores or potentially merged in research.

The three-factor ESEM model demonstrated more favorable factor distinctiveness properties, with reduced interfactor correlations: 0.725 (Vigor-Dedication), 0.605 (Vigor-Absorption), and 0.653 (Dedication-Absorption). Although the AVE values were lower in the ESEM model (Vigor = 0.370, Dedication = 0.462, Absorption = 0.522), the shared variance estimates were correspondingly reduced (ranging from 0.366 to 0.526), indicating improved factor differentiation relative to the CFA approach.

#### 3.2.2 Bifactor-CFA and bifactor-ESEM models

##### 3.2.2.1 Bifactor model analysis

To further examine the dimensionality of the UWES-S-9, bifactor models incorporating both general and specific factors were evaluated (Table 3). The bifactor-CFA model (Model 4) yielded excellent fit indices: χ^2^/df = 2.817, RMSEA = 0.060 [90% CI (0.039–0.082)], CFI = 0.988, TLI = 0.972, and SRMR = 0.022, with information criteria of AIC = 9965.452 and BIC = 10129.665. However, examination of the complementary indices (Table 4) revealed convergence issues, as evidenced by the extremely high mean standardized loading for the Dedication specific factor (λ_mean_ = 1.365), which exceeds the theoretical maximum of 1.0 and indicates model instability. This problematic parameter estimate suggests that the bifactor-CFA solution may not be trustworthy despite apparently acceptable fit indices.

**Table 3:** Comparison of model fit indices for the bifactor-CFA and bifactor-ESEM models (*n* = 498).

**Models**	**χ^2^/df**	**RMSEA (90% CI)**	**CFI**	**TLI**	**SRMR**	**AIC**	**BIC**
Model 4	2.817	0.060 [0.039–0.082]	0.988	0.972	0.022	9965.452	10129.665
Model 5	3.692	0.074 [0.055–0.093]	0.990	0.971	0.025	9983.649	10135.231

Model 4, Bifactor-CFA Model: Three S-factors (VI, DE, and AB) and one G-factor; Model 5, Bifactor-ESEM Model: Three S-factors (VI, DE, and AB) and one G-factor; G-Factor, General factor estimated as part of a bifactor model; S-Factors, Specific factors estimated as part of a bifactor model.

**Table 4:** Comparison of complementary indices for the bifactor-CFA and bifactor-ESEM models (*n* = 498).

**Models**	**G-Factor (λ_mean_)**	**S-Factors (λ_mean_)**	**ECV**	**PUC**	**ω_H_**	**ω_HS_**
Model 4	0.913	VI = 0.306, DE = 1.365, AB = 0.454	0.712	0.833	0.713	VI = 0.052, AB = 0.189, DE = 0.423
Model 5	0.690	VI = 0.266, DE = 0.322, AB = 0.457	0.789	0.667	0.827	VI = 0.024, AB = 0.041, DE = 0.149

Model 4, Bifactor-CFA Model: Three S-factors (VI, DE, and AB) and one G-factor; Model 5, Bifactor-ESEM Model: Three S-factors (VI, DE, and AB) and one G-factor; G-Factor, General factor estimated as part of a bifactor model; S-Factors, Specific factors estimated as part of a bifactor model; λ_mean_, Mean standardized factor loadings; ECV, Explained common variance; PUC, Percentage of uncontaminated correlations; ω_H_, Omega hierarchical coefficient; ω_HS_, Omega hierarchical coefficient for each subscale; VI, Vigor factor; DE, Dedication factor; AB, Absorption factor.

In contrast, the bifactor-ESEM model (Model 5) demonstrated both acceptable fit statistics: χ^2^/df = 3.692, RMSEA = 0.074 [90% CI (0.055–0.093)], CFI = 0.990, TLI = 0.971, and SRMR = 0.025, with AIC = 9983.649 and BIC = 10135.231, and theoretically plausible parameter estimates.

##### 3.2.2.2 Complementary bifactor indices

Given the convergence issues with the bifactor-CFA model (Model 4), interpretation focuses primarily on the bifactor-ESEM model (Model 5). This model presented theoretically acceptable parameter estimates (Table 4), with a general factor loading of λ_mean_ = 0.690 and balanced specific factor loadings: Vigor (λ_mean_ = 0.266), Dedication (λ_mean_ = 0.322), and Absorption (λ_mean_ = 0.457). The explained common variance (ECV = 0.789) indicated that the general factor accounted for ~79% of the common variance among items, suggesting strong evidence for a general engagement factor.

The percentage of uncontaminated correlations (PUC = 0.667) was moderate, falling below the conventional threshold of 0.70 but remaining within an acceptable range given the three specific factors structure. While this moderate PUC value suggests some caution in interpreting a perfect one-factor structure, it does not preclude the appropriateness of a primarily unidimensional interpretation. The omega hierarchical coefficient for the general factor (ωH = 0.827) was substantial and exceeded the recommended threshold of 0.70, supporting the reliability of a general engagement score. Conversely, the omega hierarchical coefficients for specific factors were negligible across all dimensions: Vigor (ωHS = 0.024), Dedication (ωHS = 0.149), and Absorption (ωHS = 0.041), indicating minimal reliable variance attributable to specific factors beyond the general factor.

### 3.3 Reliability analysis

Chi-square difference tests revealed violations of tau-equivalence assumptions across all UWES-S-9 subscales [Δχ^2^_(6)_ = 109.839, *p* < 0.001], indicating heterogeneous factor loadings. Consequently, internal consistency reliability was assessed using McDonald's omega coefficients derived from a congeneric three-factor CFA, as this approach demonstrates greater robustness to unequal factor loadings compared to coefficient alpha. The omega coefficients for Vigor, Dedication, and Absorption factors of UWES-S-9 were 0.800, 0.786, and 0.854, respectively, indicating adequate reliability. Additionally, the composite reliability (CR) values for the UWES-S-9 subscales—Vigor, Dedication, and Absorption—were 0.70, 0.79, and 0.87, respectively, reflecting acceptable levels of reliability. However, the traditional reliability estimates should be interpreted cautiously in light of the bifactor analysis results, which suggest that most reliable variance is attributable to the general factor rather than specific dimensions.

In addition to internal consistency, test–retest reliability was examined to evaluate the temporal stability of the UWES-S-9. Participants (*n* = 203) completed the scale at two time points with a 14-day interval. Results indicated moderate to good test-retest reliability for the overall UWES-S-9 [ICC = 0.771, 95% CI (0.709, 0.821)] and its subscales: Vigor [ICC = 0.745, 95% CI (0.677, 0.801)], Dedication [ICC = 0.778, 95% CI (0.718, 0.827)], and Absorption [ICC = 0.773, 95% CI (0.711, 0.823)]. These findings suggest that the UWES-S-9 demonstrates temporal stability, supporting its use as a reliable measure of academic engagement in educational settings.

As shown in Table 5, all item-corrected total correlations exceeded 0.50, providing additional descriptive support for internal consistency. Although Cronbach's alpha assumes tau-equivalence and may either underestimate or overestimate reliability when this assumption is violated, the UWES-S-9 demonstrated a high alpha coefficient in this study (α = 0.901), indicating strong internal consistency. “Alpha if item deleted” analyses also yielded consistently high values ranging from 0.886 to 0.898, suggesting that removing any single item would not improve reliability. These results, while interpreted with caution, are consistent with the omega-based findings and further suggest that the scale has good internal consistency.

**Table 5:** Reliability analysis for the UWES-S-9 (*n* = 498).

**Item**	**Subscales**	**M (SD)**	**Corrected item-total correlation**	**Alpha without item**
1. When I'm doing my work as a student, I feel bursting with energy	Vigor	3.46 (0.86)	0.600	0.895
2. I feel strong and vigorous when I'm studying or going to class	Vigor	3.35 (0.85)	0.735	0.886
3. I am enthusiastic about my studies	Dedication	3.50 (1.01)	0.712	0.887
4. My study inspires me	Dedication	3.53 (0.95)	0.672	0.890
5. When I get up in the morning, I feel like going to class	Vigor	2.60 (1.05)	0.669	0.891
6. I feel happy when I am studying intensely	Absorption	3.24 (0.98)	0.574	0.898
7. I am proud of my studies	Dedication	2.76 (1.01)	0.703	0.888
8. I am immersed in my studies	Absorption	3.32 (0.86)	0.664	0.891
9. I get carried away when I am studying	Absorption	3.10 (0.95)	0.721	0.886

### 3.4 Construct validity of the UWES-S-9

Construct validity of a measurement model can be evaluated by checking whether the fit indices have achieved the recommended threshold. The comprehensive analytical approach provided robust evidence for construct validity through multiple complementary criteria. While traditional fit indices supported the adequacy of the three-factor structure, the superior performance of ESEM models and the bifactor analysis results provided deeper insights into the construct's dimensional nature. The bifactor-CFA model, despite showing excellent fit statistics, exhibited convergence problems with implausible parameter estimates, rendering it unsuitable for interpretation. Nevertheless, the bifactor-ESEM results provided compelling evidence that the optimal structure of the UWES-S-9 is unidimensional, as the general engagement factor accounted for 79% of the common variance and minimal reliable variance attributable to specific Vigor, Dedication, and Absorption dimensions. The good model fit and strong omega hierarchical coefficient for the general factor (ωH = 0.827) support the use of a total engagement score, while the negligible specific factor reliabilities suggest limited utility of subscale scores. Although the moderate PUC value (0.667) indicates that treating the UWES-S-9 as perfectly unidimensional may introduce some parameter bias, the pattern of omega coefficients strongly favors a unidimensional interpretation over the traditional three-factor structure. The convergence of evidence from multiple analytical approaches strengthens confidence in the scale's construct validity while clarifying the predominantly unidimensional nature of the engagement construct as measured by the UWES-S-9.

### 3.5 Scale equivalence with the UWES-S-17

The results revealed a strong positive correlation between the UWES-S-9 and the original UWES-S-17 (*r* = 0.961; *p* < 0.01), indicating substantial agreement between the two versions. As detailed in Table 6, each subconstruct of the UWES-S-9 demonstrated robust correlations with its corresponding subconstruct in the original UWES-S-17. Specifically, the Vigor subconstructs showed a significant positive relationship (*r* = 0.750; *p* < 0.01), as did the Dedication subconstructs (*r* = 0.814; *p* < 0.01) and the Absorption subconstructs (*r* = 0.806; *p* < 0.01). These findings provide robust evidence of the scale equivalence with the UWES-S-17, supporting its capacity to measure the same theoretical constructs as the original scale in a more concise format.

**Table 6:** The correlations between the UWES-S-9 and the UWES-S (*n* = 498).

**Constructs**	**M (SD)**	**VI 9**	**DE 9**	**AB 9**	**Total 9**	**VI 17**	**DE 17**	**AB 17**	**Total 17**
VI 9	3.13 (0.77)	1							
DE 9	3.26 (0.82)	0.733^**^	1						
AB 9	3.22 (0.75)	0.681^**^	0.801^**^	1					
Total 9	3.21 (0.71)	0.885^**^	0.933^**^	0.908^**^	1				
VI 17	3.77 (0.84)	0.750^**^	0.854^**^	0.701^**^	0.848^**^	1			
DE17	3.33 (0.87)	0.632^**^	0.814^**^	0.605^**^	0.682^**^	0.581^**^	1		
AB 17	3.05 (0.67)	0.682^**^	0.733^**^	0.806^**^	0.813^**^	0.652^**^	0.705^**^	1	
Total 17	3.23 (0.67)	0.871^**^	0.894^**^	0.854^**^	0.961^**^	0.895^**^	0.812^**^	0.838^**^	1

^**^P < 0.01; VI 9, Vigor subscale of UWES-S-9; DE 9, Dedication subscale of UWES-S-9; AB 9, Absorption subscale of UWES-S-9; Total 9, Total of UWES-S-9; VI 17, Vigor subscale of UWES-S; DE 17, Dedication subscale of UWES-S; AB, Absorption subscale of UWES-S; Total 17, Total of UWES-S.

### 3.6 Concurrent validity of the UWES-S-9

To examine the relationship between academic engagement and academic burnout, a Pearson correlation analysis was performed. As illustrated in Table 7, the findings revealed significant negative correlations between academic engagement and academic burnout, including their respective subconstructs. Notably, a strong negative correlation was observed between overall academic engagement and academic burnout (*r* = −0.627; *p* < 0.01). Further analysis of the subconstructs showed varying degrees of negative correlations. The weakest, yet still significant, negative correlation was found between the Absorption subscale of UWES-S-9 and the Inappropriate Behavior subscale of LBS (*r* = −0.411; *p* < 0.01). Conversely, the strongest negative correlation was observed between the Vigor subscale of UWES-S-9 and the overall LBS score (*r* = −0.623; *p* < 0.01). These results suggest a negative relationship between academic engagement and academic burnout across various dimensions of these constructs, which are consistent with previous studies examining this relationship, providing substantial evidence for the concurrent validity of the UWES-S-9.

**Table 7:** The correlations between the UWES-S-9 and the LBS (*n* = 498).

**Construct**	**M (SD)**	**VI 9**	**DE 9**	**AB 9**	**Total 9**	**LM**	**IB**	**LSA**	**LBS**
VI 9	3.13 (0.77)	1							
DE 9	3.26 (0.82)	0.733^**^	1						
AB 9	3.22 (0.75)	0.681^**^	0.801^**^	1					
Total 9	3.21 (0.71)	0.885^**^	0.933^**^	0.908^**^	1				
LM	2.97 (0.71)	−0.549^**^	−0.502^**^	−0.476^**^	−0.560^**^	1			
IB	2.75 (0.77)	−0.495^**^	−0.424^**^	−0.411^**^	−0.487^**^	0.649^**^	1		
LSA	2.82 (0.65)	−0.500^**^	−0.445^**^	−0.433^**^	−0.505^**^	0.469^**^	0.470^**^	1	
LBS	2.89 (0.59)	−0.623^**^	−0.556^**^	−0.530^**^	−0.627^**^	0.905^**^	0.846^**^	0.706^**^	1

^**^P < 0.01; VI 9, Vigor subscale of UWES-S-9; DE 9, Dedication subscale of UWES-S-9; AB 9, Absorption subscale of UWES-S-9; Total 9, Total of UWES-S-9; LM, Low Mood subscale of LBS; IB, Inappropriate Behavior subscale of LBS; LSA, Low Sense of Accomplishment subscale of LBS.

### 3.7 Measurement invariance across demographic variables

Multigroup CFA was conducted to assess the measurement invariance of the UWES-S-9 across gender, academic year, and major. Results as shown in Table 8 indicated that the changes in model fit indices of the ΔRMSEA, ΔSRMR, ΔCFI, $\Delta \hat{\gamma }$, and ΔMNCI between configural and metric, and metric and scalar were no more than the cutoff values of 0.015, 0.03, 0.01, 0.008, 0.02, respectively (Chen, 2007; Kang et al., 2016), supported configural, metric, and scalar invariance, indicating that the scale's structure, factor loadings, and item intercepts are equivalent across these demographic groups. This establishes the UWES-S-9's validity for meaningful comparisons of academic engagement levels between genders and across different academic years and majors.

**Table 8:** Fit indices for measurement invariance across demographic variables (*n* = 498).

**Model**	**Invariance**	**χ^2^ (*df*)**	**χ^2^/df**	**RMSEA (ΔRMSEA)**	**SRMR (ΔSRMR)**	***Δχ*^2^ (Δ*df*)**	**CFI (ΔCFI)**	**$\hat{\gamma }$ ($\Delta \hat{\gamma }$)**	**MNCI (ΔMNCI)**
**Gender**									
1	Configural	172.509** (62)	2.782	0.060 (–)	0.054 (–)	–	0.953 (–)	0.953 (–)	0.900 (–)
2	Metric	191.990** (71)	2.704	0.059 (−0.001)	0.054 (−0.000)	19.481* (9)	0.949 (−0.004)	0.953 (0)	0.900 (0)
3	Scalar	197.320** (77)	2.563	0.056 (−0.003)	0.068 (0.014)	5.330 (6)	0.949 (0)	0.948 (−0.005)	0.889 (−0.011)
**Academic year**									
1	Configural	214.898** (110)	1.954	0.044 (–)	0.043 (–)	–	0.956 (–)	0.956 (–)	0.903 (–)
2	Metric	261.441** (128)	2.043	0.046 (0.002)	0.056 (0.013)	46.543** (18)	0.945 (−0.011)	0.956 (0)	0.906 (0.003)
3	Scalar	305.416** (158)	1.933	0.043 (−0.003)	0.061 (0.006)	43.975 (30)	0.939 (−0.006)	0.944 (−0.012)	0.879 (−0.027)
**Major**									
1	Configural	199.777** (81)	2.466	0.054 (–)	0.042 (–)	–	0.950 (–)	0.962 (–)	0.917 (–)
2	Metric	232.789** (93)	2.503	0.055 (0.001)	0.064 (0.022)	33.012** (12)	0.941 (−0.009)	0.950 (−0.012)	0.893 (−0.023)
3	Scalar	282.173** (113)	2.497	0.055 (0)	0.068 (0.003)	49.384** (20)	0.928 (−0.013)	0.941 (−0.009)	0.875 (−0.018)

*P < 0.05, **P < 0.01.

## 4 Discussion

### 4.1 Factor structure of the UWES-S-9

This study employed a comprehensive analytical framework to examine the factorial structure of the UWES-S-9, extending beyond traditional CFA to include ESEM and bifactor modeling approaches. The systematic comparison revealed important insights into the dimensional nature of this instrument.

The traditional three-factor CFA model, while demonstrating acceptable fit indices, exhibited substantial interfactor correlations (ranging from 0.653 to 0.868) and discriminant validity concerns, particularly between the Vigor-Dedication factor pair (shared variance = 0.754). These findings align with previous observations of high intercorrelations among UWES dimensions (Carmona-Halty et al., 2019; Dominguez-Lara et al., 2025; Loscalzo and Giannini, 2019; Rastogi et al., 2018; Römer, 2016; Schaufeli et al., 2006), suggesting potential challenges with the theoretical distinctiveness of the three proposed factors. The ESEM approach partially addressed these issues by allowing cross-loadings between factors, resulting in improved discriminant validity properties and superior model fit, which aligns with previous findings (Dominguez-Lara et al., 2025). The three-factor ESEM model provided superior fit statistics and more favorable factor distinctiveness properties, with reduced interfactor correlations and improved parameter estimates. This improvement suggests that allowing for cross-loadings between factors better accommodates the complex structure underlying academic engagement, consistent with recent recommendations for ESEM applications in psychological measurement (Marsh et al., 2014).

However, the bifactor analysis provided the most informative perspective on the scale's dimensional structure. While the bifactor-CFA model exhibited convergence issues (evidenced by implausible parameter estimates), which aligns with the previous study (Dominguez-Lara et al., 2025), the bifactor-ESEM model revealed theoretically coherent results. The general factor accounted for 79% of common variance, with a substantial omega hierarchical coefficient (ωH = 0.827), indicating strong evidence for a unidimensional interpretation. In contrast, the specific factors exhibited minimal unique reliable variance (ωHS ≤ 0.149), indicating that the subscale scores offer limited additional value beyond the general engagement factor. This finding aligns with the conclusions of prior studies conducted in Ecuador (Dominguez-Lara et al., 2025).

The moderate percentage of uncontaminated correlations (PUC = 0.667) indicates that while treating the scale as perfectly unidimensional may introduce some parameter bias, the evidence strongly favors a general engagement interpretation over distinct subscale usage. These findings diverge from some validation studies that have supported distinct three-factor structures (Meng and Jin, 2017; Rastogi et al., 2018; Sulla et al., 2023) while aligning with research suggesting predominantly unidimensional conceptualizations of academic engagement (Chi et al., 2023; Dominguez-Lara et al., 2025; Loscalzo and Giannini, 2019; Römer, 2016; Serrano et al., 2019). The inconsistency in reported factor structures across studies may be attributed to variations in sample characteristics, statistical methodologies, and cultural contexts, though these potential explanations require further investigation.

### 4.2 Other psychometric properties of the UWES-S-9

The second research objective of this study is to assess the internal consistency reliability, test-retest reliability, composite reliability, construct validity, its scale equivalence and concurrent validity of UWES-S-9. Findings indicate that the UWES-S-9 exhibit good internal consistency reliability, test-retest reliability, and composite reliability as well as the construct validity. This study found higher test-retest reliability coefficients for the UWES-S-9 compared to previous validation studies of UWES-S conducted in Australia and Norway (Schaufeli et al., 2006). This discrepancy may be attributed to differences in the time interval between test administrations. The current study employed a 2-week interval, while the previous study used a 1-year interval. This methodological difference underscores the importance of considering temporal factors when interpreting stability coefficients, as shorter intervals typically yield higher reliability estimates due to reduced genuine change and enhanced memory effects.

Besides that, the values of Composite Reliability (CR) for the three sub-constructs of UWES-S-9 in this study align with findings from previous research conducted in Italy (Loscalzo and Giannini, 2019; Sulla et al., 2023), India (Rastogi et al., 2018), and Spain (Serrano et al., 2019). These consistent results across different cultural contexts provide strong evidence for the scale's reliability and validity in cross-cultural research. This comparison of psychometric properties across studies and cultures underscores the robustness of the UWES-S-9 as a measure of academic engagement. However, the bifactor analysis provided critical insights that contextualize these traditional reliability estimates. The omega hierarchical coefficient for the general factor (ωH = 0.827) substantially exceeded the threshold for reliable interpretation, while specific factor reliabilities were negligible (ωHS ≤ 0.149). This pattern suggests that while the scale demonstrates overall reliability, the majority of reliable variance is attributable to a general engagement factor rather than specific dimensional components.

This study revealed a significant correlation between the UWES-S-9 and its original scale, the UWES-S, supporting its scale equivalence. This finding aligns with previous validation research conducted across multiple countries, including Australia, Belgium, Canada, Finland, France, Germany, Netherlands, Norway, South Africa, and Spain, utilizing a comprehensive international database (Schaufeli et al., 2006). The consistency of these results across diverse cultural contexts provides strong evidence that the UWES-S-9 effectively measures the same underlying constructs as the original, longer scale. This supports the scale equivalence of the UWES-S-9 as a more concise instrument for assessing academic engagement, which also enhances our understanding of the UWES-S-9's applicability and reliability across a broader range of cultural settings, reinforcing its utility as a globally relevant measure of academic engagement.

The relationship between academic engagement and academic burnout was examined in this study and the results revealed significant negative correlations between academic engagement and academic burnout, including their respective subconstructs, which is in line with the previous validation studies conducted in Korea, Italy, Australia, Belgium, Canada, Finland, France, Germany, Netherlands, Norway, South Africa, and Spain (Rastogi et al., 2018; Römer, 2016; Schaufeli et al., 2006). The cross-cultural stability of the negative correlation between academic engagement and academic burnout further verifies the concurrent validity of the UWES-S-9.

To assess measurement invariance across demographic variables, multi-group CFA was employed. The findings indicated that the UWES-S-9 demonstrates consistent measurement across various demographic groups. Specifically, the analysis supported measurement invariance for the UWES-S-9 when considering gender, academic year, and field of study. These results align with previous research findings, such as those reported by (Chi et al. 2023) and (Carmona-Halty et al. 2019). This concordance with established literature lends further support to the robustness of the present outcomes. The observed invariance suggests that the UWES-S-9 maintains its psychometric properties and measures the intended construct equivalently across these diverse demographic categories.

### 4.3 Implications, limitations and recommendations

#### 4.3.1 Theoretical and practical implications

The findings of this study not only contribute to the theoretical understanding of academic engagement but also offer practical implications for its assessment in Chinese educational settings. Theoretically, it contributed to the broader debate on the dimensionality of academic engagement and its measurement. This validation not only advances the theoretical understanding of academic engagement but also offers a more nuanced perspective on its measurement in diverse cultural settings. The comprehensive approach to validation provides a rigorous framework that can be replicated in diverse educational settings. This contribution is particularly valuable for international comparative education research, where methodologically sound cross-cultural adaptations of assessment tools are essential.

The evidence for a predominantly general factor suggests that Vigor, Dedication, and Absorption may represent manifestations of a broader engagement construct rather than distinct, separable dimensions. This finding challenges the practical utility of interpreting separate subscale scores and supports previous suggestions (Loscalzo and Giannini, 2019) to consider both total scores and subscales, though the current results more strongly favor total score interpretation. This perspective aligns with recent study emphasizing the unidimensional construct of engagement (Dominguez-Lara et al., 2025).

From a practical standpoint, these results support the use of total UWES-S-9 scores for assessing academic engagement in Chinese higher education contexts. The strong reliability of the general factor (ωH = 0.827) provides confidence in aggregate scoring approaches, while the minimal specific factor reliabilities caution against interpreting subscale scores as meaningful distinct measures. This has important implications for educational practitioners and researchers who may wish to focus on overall engagement levels rather than attempting to differentiate between specific engagement dimensions. Additionally, the demonstrated reliability and validity of the UWES-S-9 underscore its utility as a concise yet psychometrically good instrument for assessing academic engagement among Chinese undergraduate students. Notably, the scale's robust test-retest reliability enhances its applicability in longitudinal studies and interventions aimed at fostering academic engagement. The UWES-S-9's brevity makes it particularly suitable for large-scale assessments and repeated measures designs. The demonstrated measurement invariance across gender, academic year, and major enhances the scale's utility for comparative research and ensures that observed differences reflect genuine engagement variations rather than measurement issues. This psychometric property is particularly valuable for large-scale educational assessments and longitudinal intervention studies.

#### 4.3.2 Limitations and recommendations

Several limitations warrant consideration in interpreting these findings. The sample was drawn from a single Chinese province and focused on undergraduate students from normal universities, potentially limiting generalizability to other populations and educational contexts. The convergence issues observed in the bifactor-CFA model suggest that further investigation of optimal modeling approaches may be beneficial.

Future research should address these limitations through several approaches. First, replication studies across diverse educational contexts and student populations would enhance generalizability. Second, given the predominantly unidimensional findings, researchers might consider developing or adapting engagement measures that better capture theoretically distinct dimensions if such distinctions are deemed theoretically important. Third, longitudinal studies examining the stability of the factorial structure and the predictive validity of total vs. subscale scores would provide valuable insights into the scale's practical utility. Additionally, cross-cultural comparative studies employing similar comprehensive analytical approaches could illuminate whether the predominantly unidimensional structure observed here generalizes across different cultural contexts or reflects specific characteristics of Chinese student populations.

## 5 Conclusions

This study provides robust evidence for the psychometric adequacy of the Chinese UWES-S-9 while offering important insights into its dimensional structure. The scale demonstrates adequate traditional psychometric properties and measurement invariance across demographic groups. However, the comprehensive analytical approach revealed that while traditional factor analytic approaches support a three-factor structure, bifactor modeling suggests that UWES-S-9 is best regarded as a predominantly unidimensional construct. The strong reliability of the general engagement factor supports the use of total scores, while the minimal specific factor reliabilities question the utility of subscale interpretations. These findings contribute to both the theoretical understanding of academic engagement and the practical application of the UWES-S-9 in Chinese educational contexts, providing a validated instrument for measuring overall academic engagement while informing future theoretical and methodological developments in engagement research.

## Data Availability

The raw data supporting the conclusions of this article will be made available by the authors, without undue reservation.
